# Measuring the diffusion of palliative care in long-term care facilities – a death census

**DOI:** 10.1186/1472-684X-8-1

**Published:** 2009-01-16

**Authors:** Sophie Paroz, Brigitte Santos-Eggimann

**Affiliations:** 1 Institute of Social and Preventive Medicine (IUMSP), Centre Hospitalier Universitaire Vaudois and University of Lausanne, Lausanne, Switzerland

## Abstract

**Background:**

The dissemination of palliative care for patients presenting complex chronic diseases at various stages has become an important matter of public health. A death census in Swiss long-term care facilities (LTC) was set up with the aim of monitoring the frequency of selected indicators of palliative care.

**Methods:**

The survey covered 150 LTC facilities (105 nursing homes and 45 home health services), each of which was asked to complete a questionnaire for every non-accidental death over a period of six months. The frequency of 4 selected indicators of palliative care (resort to a specialized palliative care service, the administration of opiates, use of any pain measurement scale or other symptom measurement scale) was monitored in respect of the stages of care and analysed based on gender, age, medical condition and place of residence.

**Results:**

Overall, 1200 deaths were reported, 29.1% of which were related to cancer. The frequencies of each indicator varied according to the type of LTC, mostly regarding the administration of opiate. It appeared that the access to palliative care remained associated with cancer, terminal care and partly with age, whereas gender and the presence of mental disorders had no effect on the indicators. In addition, the use of drugs was much more frequent than the other indicators.

**Conclusion:**

The profile of patients with access to palliative care must become more diversified. Among other recommendations, equal access to opiates in nursing homes and in home health services, palliative care at an earlier stage and the systematic use of symptom management scales when resorting to opiates have to become of prime concern.

## Background

With an aging population, palliative care has been increasingly discussed in recent years and has developed into a separate medical specialization that differs from and is complementary to curative care. Historically, palliative care was originally intended for patients suffering from a cancer or a terminal illness. Both the definition and the objectives of such care have recently been redefined, to include patients presenting with any complex chronic disease and to offer such care early in the course of the illness [1]. Following these redefinitions, numerous development programs have been set up and training courses as well as specialized structures are now available in many countries and areas [2-7].

In Switzerland, where palliative care are included in the basket of services covered by the Swiss obligatory health insurance, the State of Vaud financed a five-year (2002–2007) palliative care development program the aim of which was to offer every patient equal access to such care, irrespective of age, pathology and place of residence. The program intended to develop palliative care for stabilised chronic patients as well as for complex intensive situations, at home, in LTC institutions as well as in hospitals and to train primary health care providers as well as specialists. The initial steps of the program were the improvement of professional training in the field of palliative care (including for LTC professionals), the establishment of a mobile palliative care teams for each region of the state, the creation of a sub-acute hospital palliative care service and the creation of an academic chair of palliative care. These first measures intended to disseminate palliative care in all care settings and places of residence.

When reviewing the results of other studies [8-11], the dissemination of palliative care across various demographic strata, various medical conditions and various stages of disease can be questioned. An important question at the present time, after years of reorganization, is to ascertain whether a wider variety of patients really does have access to palliative care. This question is a relevant public health issue, as is the need to improve the knowledge for delivering palliative care in all settings for a large range of conditions [12-15].

With this objective in mind, we set up a death census in long-term care facilities (LTC), with the aim of monitoring the frequency of various indicators of palliative care, in respect of age and gender, type of disease, place of residence, complexity of the situation (i.e. when mental disorders, usually dementia, are part of the problem) and observing the timing of palliative care (early or late on the eve of death).

## Methods

### Design and sample

The survey was conducted by the Institute of Social and Preventive Medicine at the University of Lausanne as part of the evaluation of the state initiative for promoting palliative care [16]. It analysed the circumstances of non-accidental deaths over a period of six months in beneficiaries of LTC residing in this Swiss region of 660'000 inhabitants. Two kinds of LTC structures were surveyed: a) home health services (HHS) that provide help and care, in collaboration with private primary care practitioners, to patients living at home and b) nursing homes or hospital services for chronic patients (NH) that take care of institutionalized patients. This target population was selected because it was highly susceptible to present (and die from) progressive chronic diseases and, therefore, to need palliative care early or late during the last year of life. The combination of these two types of LTC institutions also covered a wide range of patients regarding age, diagnosis and place of residence (including institutionalized and community-dwelling patients).

Every public or private NH and HHS (203 structures in total) was contacted in Spring 2006 and invited to take part in the research. Overall, the survey covered 150 LTC institutions (73.9%), including 105 NH (out of 158, 66.5%) and all HHS (45, 100%). Participating NH accounted for 4451 beds (72.1% of all LTC beds). Non-participating NH were mainly psychiatric institutions characterized by younger patients and a lower level of mortality.

### Data collection

Participating LTC institutions completed a short, anonymous questionnaire for each non-accidental death among their patients from June 15, 2006 to December 15, 2006, including those deaths that occurred in another setting (e.g. in an acute care hospital). If no death occurred in a given month, they returned a form announcing the absence of any event. One person per institution, usually a nurse, was designated to collect the questionnaires and forms, return them to the research centre on the 15^th ^of the month and respond to any questions about the records. In order to check the data, the first author systematically contacted the LTC when confronted with incomplete or contradictory data.

### Questionnaire and indicators of palliative care

The questionnaire was drawn up in the research centre, discussed with a working-group made up of NH and HHS health professionals and pre-tested on 74 fatalities. The pre-test intended to check the understanding of the items by LTC health professionals, which appeared to be good. It covered general characteristics such as age category, gender, transfers between institutions, place of death, presence of a diagnosis of cancer or recognized mental disorders that were part of the patient's situation. Four indicators of palliative care were included in the reporting form: a) recourse to a specialized palliative care service (SPCS) such as a mobile team, a call service or a hospital-based unit, b) the administration of opiates, c) the use of any pain measurement scale and d) the use of any other symptoms measurement scale. The state program had no quantifiable expectations regarding these specific indicators and their selection was guided by pragmatic reasons; they had to be clearly associated to the practice of palliative care, likely to be known by LTC professionals in charge of the deceased patient and registered in the medical file of deceased patients. Palliative care indicators were measured only for the care provided within or in collaboration with the LTC institutions. Moreover, each indicator was recorded both for the last ten days of life (late care) and for the last year of life up to the last ten days (early care). The choice of limiting *late care *to the last ten days was also guided by pragmatism because we were not in a position to decide what *late *and *early *palliative care should be in each case.

In order to avoid double counting, patients transferred between LTC structures shortly before death were counted only in the last institution in charge.

### Statistical analysis

Descriptive statistics first depicted the general characteristics of the entire population of deaths occurring in LTC. Analyses then successively compared the profile of deceased patients in NH and HHS, with and without cancer, and presenting with or without mental disorders as a component of their situation. Analyses of palliative care indicators followed the same sequence. Finally, subgroup analyses studied whether recourse to a SPCS and administration of opiates, respectively, were each associated with higher levels of other palliative care indicators. In all cases, the subpopulations were compared by means of chi-squared and Fisher's exact tests using the STATA 9 software.

## Results

Of the 1252 fatalities reported, 52 were excluded: 49 questionnaires (42 received from HHS and 5 from NH) were considered as potential duplicates related to transfers, 2 HHS cases were revealed to be accidental deaths, and in one NH case the death concerned the tenant of an apartment not covered by the survey of LTC patients.

Consequently, a total of 1200 valid death cases reported by the LTC facilities were analysed. The 105 participating NH accounted for more than half of the institutions recorded in the survey (n = 632, 52.7%). On the basis of the latest official NH statistics in Vaud we would have expected 626 cases in the participating institutions. The number of deaths reported by HHS (n = 568, 47.3%) was however lower than expected on the basis of the statistics of the HHS association in Vaud (726 cases recorded during the study period).

### General characteristics of deaths in LTC facilities

Half of the patients who died were over 85 years of age and more than 4 out of 10 were in the age category of 64–85 years. Women formed the majority, and about a third of the population was diagnosed with cancer or presented mental disorders (dementia and severe depression) (Additional file 1). The NH subpopulation of deaths had significantly higher proportions of women, very old patients and patients with mental disorders recorded at death, but a lower proportion of cancer diagnoses. Among the HHS deaths, men were more numerous than women.

Additional file 1 also shows major demographic differences between deceased LTC patients with and without a diagnosis of cancer. The male to female ratios were 1.3 when a cancer was recorded and 0.6 when no cancer was declared. Three quarters of cancer-related deaths occurred before the age of 85 years (while 6 out of 10 persons died at 85 years or older when no cancer was reported).

Reports of mental disorders in the last year of life were not related to the age or gender of the deceased LTC patients. However, they were significantly less frequent in cancer cases. Cancer was diagnosed in 19.8% of LTC patients dying with mental disorders and in 33.8% of deceased patients who did not have a mental disorder. The fact that missing values were more important regarding the presence of mental disorders than they were for other variables may be related to the difficulty of making such a diagnosis with old patients.

### Frequency of the palliative care indicators

Indicators of palliative care were more frequently positive during the last 10 days than earlier in the last year of life in all cases, with the unique exception of access to an SPCS for the NH subpopulation. The difference between late and early palliative care was more pronounced for the NH subpopulation, particularly regarding the access to opiates.

Consultation of an SPCS was more often reported for HHS patients than for NH patients in the ten days before death (p < 0.001) or earlier in the last year of life (p < 0.001). In both settings, however, the frequency of recourse to SPCS was low. The administration of opiates in the very last days of life was noticeably more often recorded for the NH patients than for the HHS patients (p < 0.001), while it was the same for both types of LTC earlier in the run-up to death. The highest frequency of recourse to pain measurement scales was observed for late care in NH (one case out of 5 versus one out of 8 in HHS, p < 0.05). Nevertheless, the use of pain or other symptom measurement scales was not standard in either setting, whether at a late or early stage in the course of the illness.

Overall, and in the two subgroups of patients with or without cancer, gender had no effect on indicators of palliative care. Age, however, was partly associated with the indicators. For the HHS subpopulation with a diagnosis of cancer, the proportion of positive indicators per age category varied significantly. It decreased in line with age, with a higher proportion of positive indicators in the youngest group (16–64 years old) and a lower proportion in the oldest group (> 85 years old). By contrast, there was no relationship between the indicators of palliative care and age in the HHS subpopulation without a cancer diagnosis, where proportions were generally low for all indicators. In NH, no significant effect of age was observed, except for early consultation of an SPCS, which was more frequent in the group of 65–85 year-old patients than in the 85+ age group, both for patients with and without a diagnosis of cancer. The small number of patients aged less than 65 precludes any interpretation of the indicators for the youngest subjects in NH.

All indicators of early and late palliative care were significantly more likely to be positive in the presence of cancer, for deceased patients in NH and to a greater extend for deceased patients in HHS (Additional file 2). By contrast, no significant difference was observed regarding the frequency of positive indicators in the presence or absence of reported mental disorders complicating the situation, overall and in both settings.

### Interaction between consultation of a specialized palliative care structure and other indicators

The hypothesis of specific care for people who had access to an SPCS was verified in both settings. Recourse to opiates, pain measurement scales or any other symptom measurement scales was much more frequently recorded in this subpopulation, whether early or late in the course of the illness. Apart from one case, all the differences were statistically significant (Additional file 3).

### Interaction between administration of opiates and other indicators

The extent to which the administration of opiates constitutes part of a palliative care approach can be investigated through the association with other indicators (Additional file 4). The frequency of access to SPCS and the use of pain or other symptom measurement scales was significantly higher in patients who received opiates both early and late in their disease.

However, in a large majority of cases, the administration of opiates did not involve the use of symptom measurement scales or the consultation of a specialized structure providing advice for palliative care. Although the administration of opiates was less frequently reported for HHS patients, it was more often registered in association with other indicators of palliative care than in NH patients.

## Discussion

Because of the importance of chronic diseases associated with multiple symptoms – cancer being only one example – the availability and dissemination of palliative care should be everyone's concern. The survey intended to check if there was evidence of a palliative care awareness among healthcare professionals and if this type of care is easily accessible to dying and sick people.

This essentially descriptive study focused on the frequency of selected indicators of palliative care regarding different groups of people. Most of the results were close to what could have been expected given the historical culture of palliative care and its usual association with terminal care and cancer. However, a number of interesting observations emerged, partly regarding the way in which the diffusion is measured and partly regarding the actual diffusion of palliative care.

### Data collection

The difference between the number of deaths reported to us by HHS and the number reported in their official statistics may be explained by accidental deaths, which were intentionally excluded from our survey but included in the statistics of the HHS association. The fact that we excluded from the HHS census the deaths of patients ultimately transferred to an NH and attributed them to the NH is another likely explanation.

### The choice of indicators

Measuring the access and dissemination of palliative care, which involves a variety of medical, social and psychological factors, is a complex undertaking and finding relevant indicators for measuring this phenomenon was even more difficult, since it involved a population of patients presenting various complex situations: the palliative care needs of patients with cancer are better defined and known by health professionals, while there is a lack of expertise as regards non-cancer patient care [17,18]. In addition, we were limited by the information systematically registered in the patient files of the LTC, which prevented us from chosing psychosocial indicators, for example.

Two elements have to be kept in mind concerning the four indicators of palliative care that were chosen. They do not claim to be exhaustive regarding the practice of palliative care and only measure part of the work done in the selected health services and area. They were designed to test the dissemination of palliative care and must therefore not be understood as indicators of quality of care: resort to an SPCS, opiates or symptom measurement scales is not justified in every clinical situation, and the survey did not purport to evaluate care provided in individual cases. The objective of the study was to measure the extend of use of palliative care through these indicators. We are satisfied that this objective was reached.

### A persistent tradition of care

Some of the results illustrated traditional views of palliative care, i.e. its association with terminal care, cancer and pain drugs [19]. Indeed, the access to palliative care appeared to be better late in the course of the illness and when cancer was also involved, while palliative care nowadays is precisely intended for patients presenting any complex chronic disease at any stage in its development. In addition, the recourse to opiates was much more frequent than all other indicators, yet palliative care must not be reduced to the administration of analgesic drugs.

### Encouraging signs of diffusion

Wherever palliative care appeared to be still associated with cancer and partly with age, gender and the presence of mental disorders had, by contrast, no effect on the access to palliative care. We expected worse access to palliative care for people presenting with mental disorders since they have specific needs [20], are more likely to experience difficulty in expressing their symptoms, may be more at risk regarding discrimination but also since it is more difficult for professionals to assess pain and symptom in these cases. This would result in a less frequent use of symptom measurement scales and a more frequent use of opiates; however, the above-mentioned indicators were not found to be dependant on the complexity of a situation characterized by mental disorders, a finding consistent with other recent Swiss results [21].

The finding of better access to palliative care when a specialized structure has been consulted accords with the literature review of Sykes and Thorns [22] and highlights the importance of training specialists and continuing to develop specific structures in order to disseminate palliative care.

### Worrying discrepancies

As mentioned previously, the characteristics of dying LTC patients who still lived at home appeared to differ from those of dying patients residing in institutions. This may explain most of the variations in the frequency of positive indicators observed for the two types of LTC. Some results may also be related to the medical culture of the LTC [23,24], especially the much higher proportion of SPCS consultations for the HHS subpopulation and the very much higher use of opiates in the last 10 days of life in the NH. However, this discrepancy regarding access to palliative care related to the place of residence is inconsistent with the affirmation of equal access to palliative care irrespective of place of residence or place of care.

The gap observed between late and early care is another matter of concern. Patients appeared to receive palliative care more often in their last ten days of life than earlier in the development of the disease. Other authors found that the use of opiates increased during the last week [25]. This observation runs counter to the wish of not limiting palliative care to terminal care.

Finally, the large proportion of patients who received opiates without any indication of the use of symptom measurement scales calls into question the sensitivity of this indicator when measuring the practice of palliative care. Without a simultaneous recourse to the systematic monitoring of the symptoms, which must be documented in the patient file in order to ensure an adequate communication between the multiple professionals involved in the care needed by many dying patients, we may wonder whether the administration of opiates can be considered as palliative care. In fact, the interpretation of the four indicators, when taken separately, is limited and, in the current state of knowledge, we lack a gold standard (such as the appropriate proportion of non-cancer deaths that would justify the prescription of an opiate) that would help to interpret their level. More than the absolute value of any individual indicator, the interaction of these indicators as well as their variations across demographic strata are more useful in providing information on the dissemination of palliative care.

## Conclusion

The observation of various discrepancies leads to several recommendations. The profile of the patients with access to palliative care must be adapted to the recent redefinition of palliative care and become more diversified. Palliative care earlier than in the last ten days of life has to be a prime concern. The recourse to opiates has to be accompanied by a more systematic monitoring of pain. More generally, the monitoring of symptoms must become widespread, whether with or without the use of drugs [26], and there should be less discrepancy in respect of access to palliative care between community-dwelling and institutionalized patients. Finally, it would be very useful to know the frequency of palliative indicators for patients presenting various complex situations, not only for patients with cancer, in other areas or countries.

## Competing interests

The authors declare that they have no competing interests.

## Authors' contributions

SP coordinated the research, collected the data, performed the statistical analysis and drafted the manuscript. BSE conceived the study and the data basis, participated in its coordination, supervised the analysis and helped to draft the manuscript. All authors read and approved the final manuscript.

## Pre-publication history

The pre-publication history for this paper can be accessed here:



## Supplementary Material

Additional file 1 **Additional file 1. **Characteristics of the reported deaths, overall, per type of LTC and according to the presence of a diagnosis of cancer. Click here for file

Additional file 2 **Additional file 2.** Frequencies of the indicators of palliative care, overall, per type of LTC and according to the presence of a diagnosis of cancer. Click here for file

Additional file 3 **Additional file 3**. Relation between the recourse to a specialized palliative care structure (SPCS) and the other indicators palliative care, per type of LTC. Click here for file

Additional file 4 **Additional file 4**. Relation between the administration of opiates and the other indicators of palliative care, per type of LTC. Click here for file
