# Supplementary material for: Measuring the diffusion of palliative care in long-term care facilities – a death census
Source: BMC Palliat Care. 2009 Jan 16;8:1. doi: 10.1186/1472-684X-8-1 (PMC2632992; doi:10.1186/1472-684X-8-1)
Supplement: Additional file 1 — Characteristics of the reported deaths, overall, per type of LTC and according to the presence of a diagnosis of cancer. [file 1472-684X-8-1-S1.pdf]

Table 1: Characteristics of the reported deaths, overall, per type of LTC and according to the presence of a diagnosis of cancer

	TOTAL		LTC				CANCER			
			NH°		HHS°°		Yes		No	
	N	%	N	%	N	%	N	%	N	%
**Gender**										
Male	508	*44.5*	210	*35.8*	298	*53.8***	185	*57.3*	295	*38.4***
Female	633	*55.5*	377	*64.2*	256	*46.2*	138	*42.7*	473	*61.6*
**Age**										
16-64 years old	87	*7.4*	21	*3.4*	66	*11.8***	47	*14.2*	37	*4.7***
64-85 years old	492	*41.7*	190	*30.7*	302	*53.9*	195	*59.1*	277	*34.8*
>85 years old	601	*50.9*	409	*66.0*	192	*34.3*	88	*26.7*	482	*60.6*
**Cancer**										
Yes	333	*29.1*	93	*15.4*	240	*44.5***	/	/	/	/
No	810	*70.9*	511	*84.6*	299	*55.5*	/	/	/	/
**Mental disorders**										
Yes	324	*30.5*	212	*36.7*	112	*23.1***	61	*20.3*	247	*34.5***
No	739	*69.5*	365	*63.3*	374	*77.0*	239	*79.7*	469	*65.5*

*= <0.05, ** = <0.001

°=nursing homes

°°=home health services
