# Supplementary material for: Measuring the diffusion of palliative care in long-term care facilities – a death census
Source: BMC Palliat Care. 2009 Jan 16;8:1. doi: 10.1186/1472-684X-8-1 (PMC2632992; doi:10.1186/1472-684X-8-1)
Supplement: Additional file 2 — Frequencies of the indicators of palliative care, overall, per type of LTC and according to the presence of a diagnosis of cancer. [file 1472-684X-8-1-S2.pdf]

Table 2: Frequencies of the indicators of palliative care, overall, per type of LTC and according to the presence of a diagnosis of cancer

INDICATORS	TOTAL						NH						HHS					
	Total		Cancer		No cancer		Total		Cancer		No cancer		Total		Cancer		No cancer	
	N	%	N	%	N	%	N	%	N	%	N	%	N	%	N	%	N	%
**SPCS**																		
Late care	64	*7.4*	40	*21.3*	24	*3.6***	18	*3.2*	6	*7.0*	12	*2.5**	46	*16.1*	34	*33.3*	12	*6.3***
Early care	99	*10.4*	73	*29.8*	26	*3.7***	38	*6.5*	20	*23.0*	18	*3.8***	61	*15.3*	53	*33.5*	8	*3.5***
**Opiates**																		
Late care	458	*53.3*	129	*69.4*	329	*48.9***	369	*63.9*	65	*74.7*	304	*62.8**	89	*32.1*	64	*64.7*	25	*13.2***
Early care	248	*26.3*	121	*50.0*	127	*18.1***	146	*26.3*	39	*44.8*	107	*22.9***	102	*26.3*	82	*52.9*	20	*8.6***
**Pain scale**																		
Late care	139	*16.3*	58	*31.5*	81	*12.1***	105	*18.5*	31	*36.1*	74	*15.3***	34	*11.9*	27	*27.6*	7	*3.7***
Early care	109	*11.6*	49	*21.4*	60	*8.5***	73	*13.0*	22	*25.9*	51	*10.7***	36	*9.6*	27	*18.8*	9	*3.9***
**Any symptom scale**																		
Late care	96	*11.3*	37	*20.4*	59	*8.8***	61	*10.8*	16	*18.6*	45	*9.4**	35	*12.4*	21	*22.1*	14	*7.5***
Early care	77	*8.3*	32	*14.1*	45	*6.4***	43	*7.7*	12	*14.3*	31	*6.5**	34	*9.1*	20	*14.0*	14	*6.1**

*= <0.05, ** = <0.001

°=nursing homes

°°=home health services
