# Supplementary material for: Measuring the diffusion of palliative care in long-term care facilities – a death census
Source: BMC Palliat Care. 2009 Jan 16;8:1. doi: 10.1186/1472-684X-8-1 (PMC2632992; doi:10.1186/1472-684X-8-1)
Supplement: Additional file 3 — Relation between the recourse to a specialized palliative care structure (SPCS) and the other indicators palliative care, per type of LTC. [file 1472-684X-8-1-S3.pdf]

Table 3: Relation between the recourse to a specialized palliative care structure (SPCS) and the other indicators of palliative care, per type of LTC

	NH				HHS			
	**SPCS +**		**SPCS -**		**SPCS +**		**SPCS -**	
	N	%	N	%	N	%	N	%
**Late care**								
Opiates	19	*90.5*	359	*62.9**	38	*79.2*	58	*23.3***
Pain scale	10	*47.6*	100	*17.6**	18	*40.9*	18	*7.2***
Any symptom scale	5	*23.8*	56	*9.9*	16	*36.4*	20	*8.1***
**Early care**								
Opiates	26	*70.3*	118	*22.6***	35	*70.0*	59	*18.2***
Pain scale	54	*46.9*	15	*10.3***	16	*38.1*	18	*5.5***
Any symptom scale	9	*26.5*	33	6.3**	11	*26.8*	19	*5.9***

*= <0.05, ** = <0.001

°=nursing homes

°°=home health services
