# Supplementary material for: Measuring the diffusion of palliative care in long-term care facilities – a death census
Source: BMC Palliat Care. 2009 Jan 16;8:1. doi: 10.1186/1472-684X-8-1 (PMC2632992; doi:10.1186/1472-684X-8-1)
Supplement: Additional file 4 — Relation between the administration of opiates and the other indicators of palliative care, per type of LTC. [file 1472-684X-8-1-S4.pdf]

Table 4: Relation between the administration of opiates and the other indicators of palliative care, per type of LTC

	NH				HHS			
	Opiates +		Opiates -		Opiates +		Opiates -	
	N	%	N	%	N	%	N	%
**Late care**								
SPCS	19	*5.0*	2	*0.9**	38	*39.6*	10	*5.0***
Pain scale	95	*25.1*	16	*7.5***	34	*37.4*	2	*1.0***
Any symptom scale	57	*15.1*	6	*2.9***	26	*28.9*	11	*5.6***
**Early care**								
SPCS	26	*18.1*	11	*2.7***	35	*37.2*	15	*5.3***
Pain scale	54	*37.0*	20	*4.9***	31	*35.2*	5	*1.7***
Any symptom scale	20	*13.9*	22	*5.3**	21	*25.0*	11	*3.9***

*= <0.05, ** = <0.001

°=nursing homes

°°=home health services
